# Cystic fibrosis improves COVID-19 survival and provides clues for treatment of SARS-CoV-2

**DOI:** 10.1007/s11302-021-09771-0

**Published:** 2021-05-10

**Authors:** Edward H. Abraham, Guido Guidotti, Eliezer Rapaport, David Bower, Jack Brown, Robert J. Griffin, Andrew Donnelly, Ellen D. Waitzkin, Kenon Qamar, Mark A. Thompson, Sukumar Ethirajan, Kent Robinson

**Affiliations:** 1 Saint Francis Health System, Tulsa, OK USA; 2 Kansas City Urology Care, Kansas City, KS USA; 3 Department of Molecular and Cell Biology, Harvard University, Cambridge, MA USA; 4 ATP Therapeutics, LLC, Cambridge, MA USA; 5 Horizon Medical Services, Tamarac, FL USA; 6 St. John Medical Center, Tulsa, OK USA; 7 University of Arkansas for Medical Sciences, Little Rock, AR USA; 8 Boston, MA USA; 9 Bayer GU Oncology, St. Louis, MO USA

**Keywords:** ATP, Adenosine triphosphate, COVID-19, SARS-CoV-2, Cystic fibrosis, CF, CFTR, Pannexin, Coronavirus, Adenosine, Cytokine storm

## Abstract

Systemic pools of ATP are elevated in individuals homozygous for cystic fibrosis (CF) as evidenced by elevated blood and plasma ATP levels. This elevated ATP level seems to provide benefit in the presence of advanced solid tumors (Abraham et al., Nature Medicine 2(5):593–596, 1996). We published in this journal a paper showing that IV ATP can elevate the depleted ATP pools of advanced cancer patients up to levels found in CF patients with subsequent clinical, biochemical, and quality of life (QOL) improvements (Rapaport et al., Purinergic Signalling 11(2): 251–262, 2015). We hypothesize that the elevated ATP levels seen in CF patients may be benefiting CF patients in another way: by improving their survival after contracting COVID-19. We discuss here the reasoning behind this hypothesis and suggest how these findings might be applied clinically in the general population.

The elevated blood and extracellular adenosine triphosphate (ATP) levels seen in cystic fibrosis (CF) patients may explain why some solid tumors expressing purine receptors are largely absent from CF patients; malignant melanoma is one such example. The elevated ATP levels activate the P2 receptors on the malignant cells with subsequent apoptosis and autophagy [6, 52]. In retrospect, this finding should not be surprising in view of the work of Rapaport who demonstrated that intraperitoneal (IP) administration of ATP inhibited the growth of tumors implanted into mice [37, 38]. In view of these findings, we conducted a phase II clinical trial of intravenous ATP infusion in human patients with various solid tumors. These infusions were tolerated uneventfully and produced clinical, biochemical, and quality of life improvements in some malignancies. Metastatic hormone refractory prostate cancers upregulate purinergic receptors and as a result, the addition of oral ATP to palliative agents such as bisphosphonate- chelated samarium-153 (Quadramet) dramatically reduces the prostate cancer biomarker PSA over that seen with use of the radioisotope alone [25, 30, 34, 39, 41].

In addition to protection against certain tumor cell lines, CF also appears to provide enhanced survival against SARS-CoV-2. Two recent publications—both available in late April 2020—provide important findings concerning the effect of the COVID-19 pandemic on the cystic fibrosis (CF) population worldwide. Colombo et al. present important information about the infection in the CF^1^ population in the COVID-19 hotspot of Lombardia, Italy, as well as elsewhere in Europe [14]. Cosgriff et al. present a more detailed evaluation of the effects of COVID-19 in the cystic fibrosis population in eight countries on three continents [16]. Further commentary on this phenomenon is found in [33, 40] and [45].

To more fully understand the ramifications of the two data points of Colombo et al., we constructed Table 1. This table shows that the incidence of clinically detectable COVID-19 resulting from coronavirus 2 (SARS-CoV-2) infections in Italian CF individuals appears to be 1.43 times higher than in the non-CF population in Lombardia, while the incidence of COVID-19-specific mortality in the Italian CF population is zero. The presence of a reduced mortality rate despite an increased infection rate is truly unexpected and puzzling.^2^ While the details of these findings may change with rapid improvements in detection and calculated exposure based on blood antibody titers and other assays, this data suggests that CF patients have an increased risk of contracting COVID-19 mitigated by a remarkably increased chance of survival following exposure. It is possible of course that some of the survival advantage shown in these CF patients may be due to the younger age of this group as compared to the general population; however, no age- matched population cohort could possibly have a better survival rate than the 100% survival seen in the CF patients with COVID-19.

**Table 1:** A characterization of the COVID-19 infection in Lombardia from its beginning—taken as 21 FEB 2020—until 31 MAR 2020

Populaton	Population size	# Infected	Incidence	# Deaths	Death rate
Italy ± CF	60 360 000	190 000	314	25 549	42.3
Lombardia, - CF	10 058 994	70 155	697	12 940	128.6
Lombardia, + CF	1 006	10	994	0	0

Incidence refers to those first manifesting COVID-19 during this time period. “Deaths” (“death rate”) are COVID-19-specific deaths (death rates). The incidence and death rate are given per 100 000 people of the indicated population group. The negative vs. positive signs indicate the absence vs. presence of clinical CF, respectively; the ± sign indicates the total population (i.e., including those with and without CF). Data was calculated from Colombo et al. [14] and other publications. The total number of CF patients in Lombardia was calculated based on the CF prevalence in Lombardia of 10.0 per 100 000 people as published in [20]

Recent data published by the *Cystic Fibrosis Registry Global Harmonization Group* seems to corroborate this conclusion [16]. This group reported on the outcome of 40 CF patients with SARS-CoV-2 infection located in eight participating countries and concluded that the clinical course of these patients was “better than initially predicted.” Remarkably, there were no deaths in these patients as of the time of publication. This is illustrated in Table 2. This improved survival is remarkable since CF patients begin the infection with a compromised state of respiratory function [3, 5, 6].

**Table 2:** A characterization of the worldwide COVID-19 pandemic

Country	Population size	# Deaths	Death rate	Death rate
	(millions)		CF ±	CF +
Australia	24.99	97	0.4	0
Belgium	11.46	8 415	73.4	0
France	66.99	25 897	38.7	0
Germany	83.02	7 392	8.90	0
Ireland (Republic)	4.904	1 403	28.6	0
Netherlands	17.28	5 288	30.6	0
United Kingdom (UK)	66.65	30 615	46.0	0
United States (US)	328.20	76 513	23.3	0

Deaths (death rates) are COVID-19-specific deaths (death rates). Death rates are per 100 000 people. CF + means patients positive for cystic fibrosis. CF ± indicates general population (i.e., those with CF and those without CF). Data from Cosgriff et al. [16]

CFTR gene mutations have been isolated from the dental and skeletal remains of ancient Europeans using modern DNA analytic tools, demonstrating that the CF mutation extends far back into distant antiquity.^3^ Homozygous CFTR gene mutations result in clinical CF which universally resulted in death during childhood until the mid twentieth century. Heterozygous carriers of CFTR gene mutations do not develop clinically significant CF. Any possible survival benefits of the CFTR gene mutations in the general population were thus conferred by their presence in the heterozygous CFTR gene mutation carriers. Postulated benefits of a mutated CFTR protein gene includes improved ability to survive after infection with various microbes; as mentioned above, there is evidence for CF carrier state benefit against cholera, typhoid, and tuberculosis.^4^ Survival benefits have presumably led to the current high prevalence of CFTR mutations present in the Caucasian population; approximately one out of every 20 to 25 Caucasian individuals is at least heterozygous for a CFTR protein gene mutation [8, 10, 32, 53]. It now appears that the homozygous CFTR gene mutation is demonstrating a survival benefit against a new class of microbial agent (i.e., SARS-CoV-2) with a potentially new and unknown mechanism of action. Whether heterozygous carriers of the CFTR gene mutation are also protected (as seen in the previously mentioned bacterial infections) is an open question.

CF is associated with increased viscosity of mucous of the airways and other organs. From Table 1, it is evident that the thick CF mucous does not impede SARS-CoV-2 viral entry into the airway cells and the establishment of COVID-19. CF is also associated with abnormal chloride and electrolyte abnormalities as demonstrated by the chloride sweat test. The laboratory of Abraham et al. was the first to investigate the association between CF and abnormal ATP and purine transport [3, 5, 6, 27]. They demonstrated that the CFTR protein is associated with ATP transport to the extracellular surface of epithelial cells. It now appears that CFTR controls the transmembrane release of ATP through the pannexin-1 membrane bound protein in both RBCs and epithelial cells [44]. In normal and CF physiology, the level of extracellular ATP is also controlled by CD39 and CD73, which dephosphorylate ATP and produce extracellular adenosine. Extracellular ATP and adenosine control a variety of epithelial functions through interaction with the P1 (adenosine) and P2 (ATP) purinergic receptors. Extracellular adenosine returns to the intracellular space via adenosine transporters resulting in renewed intracellular ATP and adenylate levels; adenosine may also be deaminated by the adenosine deaminase (ADA) [49]. Figure 1 presents an overview of the systemic distribution and metabolism of ATP which is especially relevant to our conceptual understanding of the CF phenotype.

**Fig. 1:**
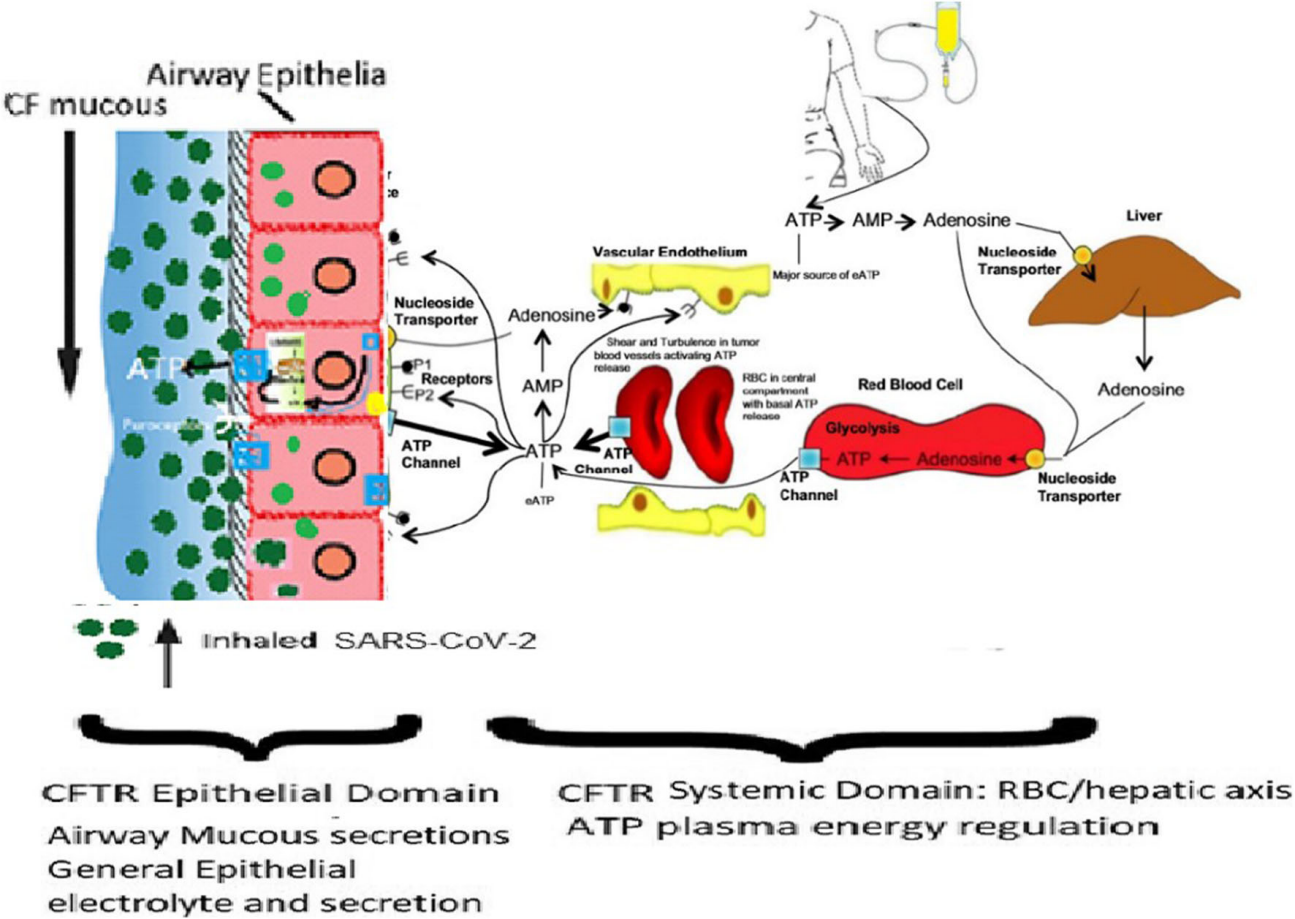
A schematic of CFTR-associated ATP transmembrane pathways including CFTR-associated pannexin-1-mediated ATP release channels. CFTR distribution can be divided between CFTR in epithelial apical and basolateral membranes and CFTR with systemic distribution especially in erythrocyte membranes. The former distribution modulates epithelial secretions including airway mucous and sweat duct sweat. Epithelial surface ATP modulates mucous composition and ciliary beat frequencies. ATP release along the vascular endothelial surfaces is an important role in this aspect of COVID-19 pathophysiology [29]. CF individuals have approximately twice the RBC and plasma ATP levels as the non-CF general population. A major source of extracellular ATP (eATP) is the infused or orally administered ATP, which is delivering ATP directly into the blood plasma compartment. After a short infusion time, the elevated eATP pools induce higher activities of catabolic ecto-enzymes. At the termination of a continuous intravenous infusion of ATP, the majority of the exogenously administered ATP is sequestered within the erythrocytes and is ultimately stored in liver hepatocytes. In phase II clinical trials, we have been able to safely bring the ATP levels of elderly patients with a variety of stage IV solid tumors up to the CF plasma and CF RBC ATP levels with resulting significant clinical and laboratory improvements. This is similar to the situation encountered in the COVID-19 patients with oral ATP supplementation

In view of these unusual findings, we endeavored to understand the physiological factors in the CF disease state that mitigate COVID-19 morbidity. While many potential factors could play a role (including whatever factors in CF patients that provide benefit against diseases such as cholera, typhoid and tuberculosis), we eventually narrowed our investigation to focus on two promising candidates—vitamin D and ATP—whose in vivo levels can be manipulated.^5^ Recent evidence suggests that elevated vitamin D levels offer some survival benefit against COVID-19 [28]. However, CF patients have deficiencies in fat-soluble vitamin (A, D, E, K) absorption and vitamin D levels are intrinsically lower in the CF population than in the general population [12]. For example, Abraham et al. reported a patient with vitamin D deficiency presenting with rickets subsequently found to be suffering from CF [7]. As opposed to vitamin D levels, however, systemic levels of ATP are intrinsically elevated in CF patients. We hypothesize that this is the crucial factor promoting improved survival in view of ATP’s central role in both intra-cellular metabolism and extracellular signaling. We now attempt to give reasons why this hypothesis is more than just an ad hoc belief, i.e., why this hypothesis makes rational sense. In defending this hypothesis, we make the preliminary observation that the function of ATP in the human body is exceedingly complex; ATP has many metabolic functions and participates in multiple pathways. Despite the truthfulness of this observation, there are certain simple facts which support the hypothesis. These facts are as follows.

Systemic, red blood cell (RBC) and blood plasma ATP pools decline with increasing age and independently with chronic diseases such as cancer, heart disease, and diabetes mellitus. This is probably secondary to the general decline in both the number of cellular mitochondria and in the quality of mitochondrial DNA. Processes such as exposure to ionizing radiation can accelerate the rate of mitochondrial DNA damage and this can by itself cause diminished mitochondrial ATP production. Mitochondria are destroyed in the process of mitochondrophagy, the cellular removal of damaged or defective mitochondria secondary to the associated buildup of mitochondrial DNA damage [9, 18, 19, 36, 48]. This systemic decline in ATP is also seen in CF patients with increasing age and chronic disease; however, when compared with age- and morbidity-matched non-CF controls, CF patients have higher levels of blood and systemic ATP. The discovery of the CFTR gene mutation in CF suggested that this mutation causes the mitochondria of CF patients to increase mitochondrial ATP production. Although not directly measuring mitochondrial ATP levels, the work of Feigel and Shapiro in the 1980s suggested that mitochondria have important roles in cystic fibrosis [42]. For example, they demonstrated that the enzyme NADH dehydrogenase plays a role in CF mitochondrial O_2_ utilization and analyzed mitochondrial O_2_ consumption in the presence and absence of rotenone inhibition. They demonstrated that oxygen consumption was greatest in the homozygous mutated CFTR phenotype, intermediate in the heterozygous CFTR phenotype and lowest in the wild type CFTR phenotype. Recent studies by Ballard and her group using myocytes showed that CFTR modulates plasma membrane pannexin-1 mediated ATP channel ATP release. In these studies, mild acidosis increased CFTR mediated bicarbonate influx through the plasma membrane. Cytoplasmic bicarbonate activated the mitochondria to release ATP and cytochrome C into the cytoplasm. Cytoplasmic cytochrome C activated the plasma membrane pannexin-1 opening which in turn resulted in ATP release to the extracellular surface. Thus, there is a causal linkage between CFTR presence in the plasma membrane and mitochondrial ATP production and plasma membrane ATP release [43, 51].

In addition to the general decline of ATP levels with age [36], other mechanisms working to decrease ATP levels may be present in COVID-19. There is substantial evidence that infection with SARS-Cov-2 is associated with blood clotting in the lungs, brain, and elsewhere [31, 56]. SARS-Cov-2 entry into local vascular endothelial cells appears to serve as a nidus for clot formation. It seems likely that the micro-clots in the lung capillary endothelium result in low Pa O_2_ with subsequent reduced local oxygenation of hemoglobin and decreased local tissue metabolic ATP production. Also, viral infections in general may deplete ATP levels. After viruses such as SARS-CoV-2 set up a successful infection state, they begin to commandeer ATP production to replicate virus particles; the adenosine in many ATP molecules is incorporated into virus particles.

But are these mechanisms causing depletion of ATP levels clinically significant? We suggest that they are vitally important. Among other functions, ATP serves as an energy carrier and signalling molecule. The human body contains about 0.1 mole of ATP, each molecule of which gains and releases energy during phosphorylation-dephosphorylation processes repeated many thousands of times each day. In fact, the human body recycles an amount of ATP each day roughly equal to its own weight. If each ATP molecule undergoes recycling so many times, is the absolute amount of ATP in the body really important? Could not increased recycling compensate for a smaller than normal amount of ATP? This question seems to require a negative answer due to the finite time required for each cycle of phosphorylation-dephosphorylation. There must be a minimum amount of ATP required to maintain homeostasis. Depletion of the systemic ATP levels below this absolute minimum prevents ATP from participating in metabolic pathways needed to distribute energy throughout the body and from participating in pathways important to the production of an immune response. With adequate ATP, an effective immune response usually develops in 4 to 6 weeks after infection with SARS-CoV-2. With depleted ATP levels, an effective immune response may not be produced. Furthermore, the compromised energy pathways may lead to fatigued respiratory muscles and impaired respiration.

In summary, CF patients may have a protective advantage in COVID-19 due to the presence of elevated ATP levels. Conversely, the ageing general population without comorbidities and even more so the ageing population with comorbidities are at great risk of morbidity and death due to ATP depletion already present at the time of viral inoculation. This is the basic reason that ATP supplementation of the general population (i.e., those without CF)—especially during the first few critical weeks of SARS-CoV-2 infection—may lead to a survival benefit. We hypothesize that ATP supplementation up to the levels seen in CF patients would mimic the CF state and provide the same level of protection seen in CF patients. Low Pa O_2_ levels, acidosis, and shear stresses are known triggers for the release of RBC ATP. Since all of these factors are present in the vicinity of microclots, supplemental ATP may aid tissue regeneration around the microclots.

Remdesivir, the antiviral agent that has demonstrated benefit in patients with severe COVID-19, is effective because it functions as an adenosine nucleoside triphosphate analog. Remdesivir is intracellularly metabolized to an active metabolite that interferes with the action of viral RNA-dependent RNA polymerase causing a decrease in viral RNA production. Remdesivir does not appear to have adverse effects on mammalian ATP metabolising enzyme systems. The presence of remdesivir results in the arrest of RNA synthesis in SARS-CoV-2. This arrest occurs after incorporation of three additional nucleotides. One can envision the benefit of administering oral or IV ATP along with remdesivir, the former replenishing mammalian host depleted ATP stores while remdesivir blocks ATP depletion by preventing access to the SARS-CoV-2 ATP metabolizing enzymatic machinery described above.

However, ATP supplementation needs to be carefully monitored and given under the care of a physician. It is not true that “If some ATP is good, even more ATP is better.” Too much ATP may result in precipitation of adenosine in blood vessels similar to the phenomenon of calcium deposition seen in certain patients undergoing dialysis (i.e., calciphylaxis). Adenosine is further metabolized to uric acid. Elevated uric acid concentrations likewise lead to precipitation and can lead to uric acid deposits in joints resulting in symptoms of gout. Precipitation in the heart, tissue and vasculature can lead to morbidity and increased mortality. It is notable that as the lifespan of CF patients continues to increase, symptoms of elevated uric acid and gout are emerging [13, 23, 26, 54].

While as noted above, blood ATP levels show a monotonic decrease with age within the general population and CF individuals, uric acid blood levels demonstrate a monotonic increase with age. These temporal blood metabolite changes are thus intrinsically coupled. Short-term boosting of blood ATP with oral ATP administration in presence of COVID-19 appears to be life preserving but prolonged administration of oral ATP is not recommended.

We first began investigating ATP supplementation dose levels after we observed transplasma membrane ATP release associated with the presence of the CFTR gene mutation. In experiments with CFTR knockout animals, the offspring of heterozygous CFTR animal matings were randomized to receive daily IP ATP or control IP phosphate buffered saline (PBS). The animals were observed for well over a year and at death genotypes determined by tail snip DNA analysis. Kaplan-Meier survival analysis showed the surprising result that mortality was greatest in the CF mice receiving ATP supplementation and lowest in the CFTR wild type receiving PBS IP injections. Analysis of blood ATP levels correlated directly with these genotypic and ATP supplementation parameters [5].In view of these findings, we recommend the following:

1. Elderly patients at risk for COVID-19 and patients with comorbidities at risk for COVID-19 should be started on low dose ATP (i.e., 400 to 800 mg daily).
2. If COVID-19 positivity develops in these two high risk groups, the ATP supplementation should be increased from 1200 to 1600 mg per day.
3. After clearance of the SARS-CoV-2 agent, the level of ATP should be reduced and discontinued after 1 or 2 months.

In summary, our recommendation is to begin low- dose ATP supplementation in elderly individuals at risk for SARS-CoV-2 exposure, to increase the ATP dose if infection is confirmed by COVID-19 assay, to reduce the level of supplemental ATP after clearance of the SARS-CoV-2 virus and finally, to discontinue supplementation altogether after a reasonable length of time.

Since deterioration of pulmonary, renal, and gastrointestinal epithelia are characteristics of COVID-19, we now suggest implementing trials of ATP supplementation designed to prevent organ failure in these patients. We previously conducted studies administering high-dose oral ATP to athletes and advanced cancer patients resulting in improved athletic performance and cancer patient survival [4, 24, 35, 39]. This approach is uncomplicated and easily applied in the setting of SARS-CoV-2-infected individuals who are not yet in intensive care and has the potential to reduce rates of hospitalization and intubation. For COVID-19 individuals who are critically ill and require intensive care, intravenous ATP infusion as described in our phase II study of cancer patients could be given [39, 41]. ATP therapy could potentially avoid the need for intubation and ventilation of SARS-CoV-2-infected individuals, thus providing time for other agents and the immune system to engage and clear the viral infection.

We now report short-term results of a non-randomized trial of ATP supplementation for treatment of clinically significant COVID-19 and for prevention of clinically manifested COVID-19 at the four institutions listed in Table 3; these institutions are listed along with their geographic location and a reference number which will be used to circumvent continual repetition of their names. (Thus, e.g., Brookdale Senior Living will be referred to as facility 2.) Reporting the results of this non-randomized trial has been approved by an institutional review board (IRB)^6^.

**Table 3:** The names and location of four institutions in which ATP supplementation was given for COVID-19

ID number	Institution name	City	State
1	Southern Hills Assisted Living	Tulsa	Oklahoma
2	Brookdale Senior Living	Tulsa	Oklahoma
3	Emerald Care Center Claremore	Claremore	Oklahoma
4	Gracewood Health and Rehab	Tulsa	Oklahoma

Facilities 1 and 2 began experiencing the rapid and insidious onset of COVID-19 in late April 2020. On 07 MAY 2020, we began offering oral ATP nutritional supplementation to COVID-19-positive patients in these two facilities. Patients were given free choice to either accept or reject ATP which was clearly documented with written consent; no randomization was involved. ATP doses ranged from 400-mg capsules four times per day in the younger patients at facility 1 to three 400-mg capsules three times per day for the older patients in facility 2. In both facilities, ATP was taken for at least a week after confirmation of SARS-CoV-2 infection. ATP also was offered in powder form to be mixed with food or drink at 450 mg per dose.

Using a protocol similar to those used in facilities 1 and 2, ATP supplementation was begun in facility 3 during the week of 03 AUG 2020. A slightly different approach was used in facility 4. In facility 4, all inpatients—regardless of COVID-19 status—were begun on a lower dose of ATP supplementation on 15 JUL 2020. This dose was increased in the event of COVID-19 positivity. Thus, at facilities 1, 2, and 3, ATP supplementation was given only to COVID-19-positive patients. In facility 4, low-level ATP supplementation was offered to all patients; the amount of supplemental ATP was increased if subsequent testing confirmed the presence of COVID-19. The remarkable retrospective findings of this study are summarized in Tables 4, 5, and 6.

**Table 4:** A snapshot of COVID-19-specific death rates in facilities 1 and 2 as of 06 JUN 2020

Population	ATP status	Average age	# Patients	# deaths	Death rate (%)
US, all ages	− ATP				3.0 ± 2.0
US, age > 70	− ATP				11.5 ± 3.5
Facility 2	− ATP	82.8	12	12	100
Facility 2	+ ATP	87.1	14	0	0
Facility 1	− ATP	74.7	7	7	100
Facility 1	+ ATP	68.4	25	0	0

Death rates are stratified according to “ATP status,” i.e., presence or absence of ATP supplementation. The phrase + ATP indicates patients receiving ATP supplementation while − ATP indicates patients not receiving supplemental ATP. COVID-19-specific death rates for the US general population and US citizens over the age of 70 years are shown for comparison

**Table 5:** A snapshot of COVID-19-specific death rates in facility 3 as of 09 SEP 2020

Population	ATP status	Average age	# Patients	# deaths	Death rate (%)
US, all ages	− ATP				3.0 ± 2.0
US, age > 70	− ATP				11.5 ± 3.5
Facility 3	− ATP	81.5	22	10	45.4
Facility 3	+ ATP	77.9	28	1 *	3.5

Death rates are stratified according to “ATP status,” i.e., presence or absence of ATP supplementation. The phrase + ATP indicates patients receiving ATP supplementation while − ATP indicates patients not receiving supplemental ATP. COVID-19-specific death rates for the US general population and US citizens over the age of 70 years are shown for comparison. * Note that the only patient who died in the supplemental ATP group was diagnosed with COVID-19 on 11 AUG 2020, started on ATP on 13 AUG 2020 and died of COVID-19 shortly thereafter after only 3 doses of ATP

**Table 6:** A snapshot of COVID-19- specific death rates in facility 4 as of 09 SEP 2020

Population	ATP status	Average age	# Patients	# deaths	Death rate (%)
US, all ages	− ATP				3.0 ± 2.0
US, age > 70	− ATP				11.5 ± 3.5
Group 1	− ATP	76.7	5	5	100
Group 2	+ ATP	69.4	61	0	0
Group 2a	+ ATP	67.3	34	0	0
Group 2b	+ ATP	72.0	27	0 *	0 *

Death rates are stratified according to group. Group 1 did not receive supplemental ATP. Group 2 initially had no COVID-19-positive individuals and all members of this group were started on prophylactic supplemental ATP at a low dose rate. Group 2 was subsequently divided into two groups—2a and 2b. Members of group 2a remained free of COVID-19 infection and continued supplemental low dose ATP. Members of group 2b became COVID-19 positive and subsequently received ATP at a higher dose. One member of group 2b died but her death was not attributed to COVID-19 by the facility’s staff. She was 84.4 years old and had multiple comorbidities including CHF, bradycardia, DM, hyperlipidemia, acute renal injury, and dementia. She died unexpectedly 32 days after her SARS-CoV-2 formal diagnosis and after completing a course of high-dose ATP. At the time of her death, she was functioning at her baseline level

In facilities 1 and 2, all patients who declined ATP died of COVID-19 while all patients accepting oral ATP were alive and recovering 2 months after the initiation of supplemental ATP. The two initial groups in each facility (presence or absence of ATP supplementation) were comparable in terms of age and comorbidities. From the initial 13 patients diagnosed with COVID-19 in facility 1 by 08 MAY 2020, six patients with average age 75.0 years chose to start ATP while the remaining seven with average age 74.7 years chose not to take ATP. Because of the visible developing benefit conferred by ATP, all subsequent COVID-19 patients at the facility 1 chose to begin taking ATP at the time of their diagnoses. All six patients who initially chose ATP were still alive and five of the six were COVID-19 free by 01 JUL 2020. At facility 2, all of the patients receiving ATP started taking ATP on the same day (27 MAY 2020) following confirmation of viral infection. All of the facility 2 patients taking ATP were alive as of 01 JUL 2020. Because all patients taking ATP were elderly with multiple co-morbidities (including diabetes and heart disease), it would be expected that their death rate should be worse than that of the general population of individuals over 70 years of age. In facility 3, all COVID-19 patients taking supplemental ATP survived except for one patient with extenuating circumstances as explained in Table 5.

In accordance with our initial research proposal that was closely evaluated and certified by an IRB, we have implemented ATP supplementation in additional COVID-19 positive nursing homes in eastern Oklahoma; as of the time of the final writing of this paper (29 DEC 2020), over 100 COVID-19-positive patients in these nursing homes have received oral ATP with excellent results. We admit the presence of some selection bias and possible placebo effect and are proceeding to implement controlled randomized trials of ATP supplementation. Pending results of such trials, we recommend that oral ATP be considered under a physician’s supervision at the first indication of SARS-CoV-2 infection in view of its great potential benefit and minimal risk.

It is interesting to note that three other research groups have proposed strategies to improve aspects of the immune response in the face of COVID-19 based on hypotheses in which ATP plays a role [1, 46, 49]. The P2X7 receptor is an ATP-gated large multi-functional channel. A recent paper by di Virgilio et al. [49] postulated blocking the P2X7 receptor to decrease the cytokine storm seen in a subset of COVID-19 patients. It is possible to consider giving COVID-19 patients experiencing cytokine storm a P2X7 blocker along with supplemental ATP to keep the putative pathway postulated by di Virgilio et al. in check while simultaneously letting these patients experience the systemic benefits of ATP pool replenishment. Taghizdeh- Hesary and Akbari argue that depleted ATP levels need to be augmented in COVID-19 patients and proposed smoking cessation, exercise, and diet to accomplish this goal [46]. Abouelkhair argues that conversion of ATP to adenosine needs to be reduced in COVID-19 patients because of the anti-inflammatory receptor A2AR [2]. Our approach focuses on the direct replenishment of systemic ATP in order to stabilize the ATP depletion found in COVID-19 infected individuals. The other targeted approaches may have roles in individuals experiencing severe cytokine storm and as additional therapy after ATP supplementation.

It is widely recognized that IV administration of ATP results in elevated blood levels. There has been some controversy over the actual ATP levels achieved after oral ATP administration. Dagnelie was unable to detect an increase in blood ATP after oral ATP administration. This is in contrast to the results of Abraham. Dagnelie’s inability to detect an increase is probably due to two reasons: use of an assay that was not as sensitive as the luciferase assay used by Abraham and also use of enteric coated ATP [39]. Following the study of low- dose enterically coated ATP administered to athletes, we performed a small study within the lab to determine if higher doses of enterically coated ATP could be detected in the blood [24]. Figure 2 shows the results and compares the changes in blood ATP following the IV ATP with changes in blood pool ATP following oral 800 mg enterically coated ATP [39]. Fluctuations in the blood level of ATP following oral administration is consistent with the systemic distribution of the administered ATP. The lower levels achieved compared to the IV ATP provide a good safety threshold for orally administered ATP. Furthermore, in the current work and following the findings of Purpura et al., we have been using non-enterically coated capsule ATP at 400 mg per capsule and also ATP administered as ATP powder [35].

**Fig. 2:**
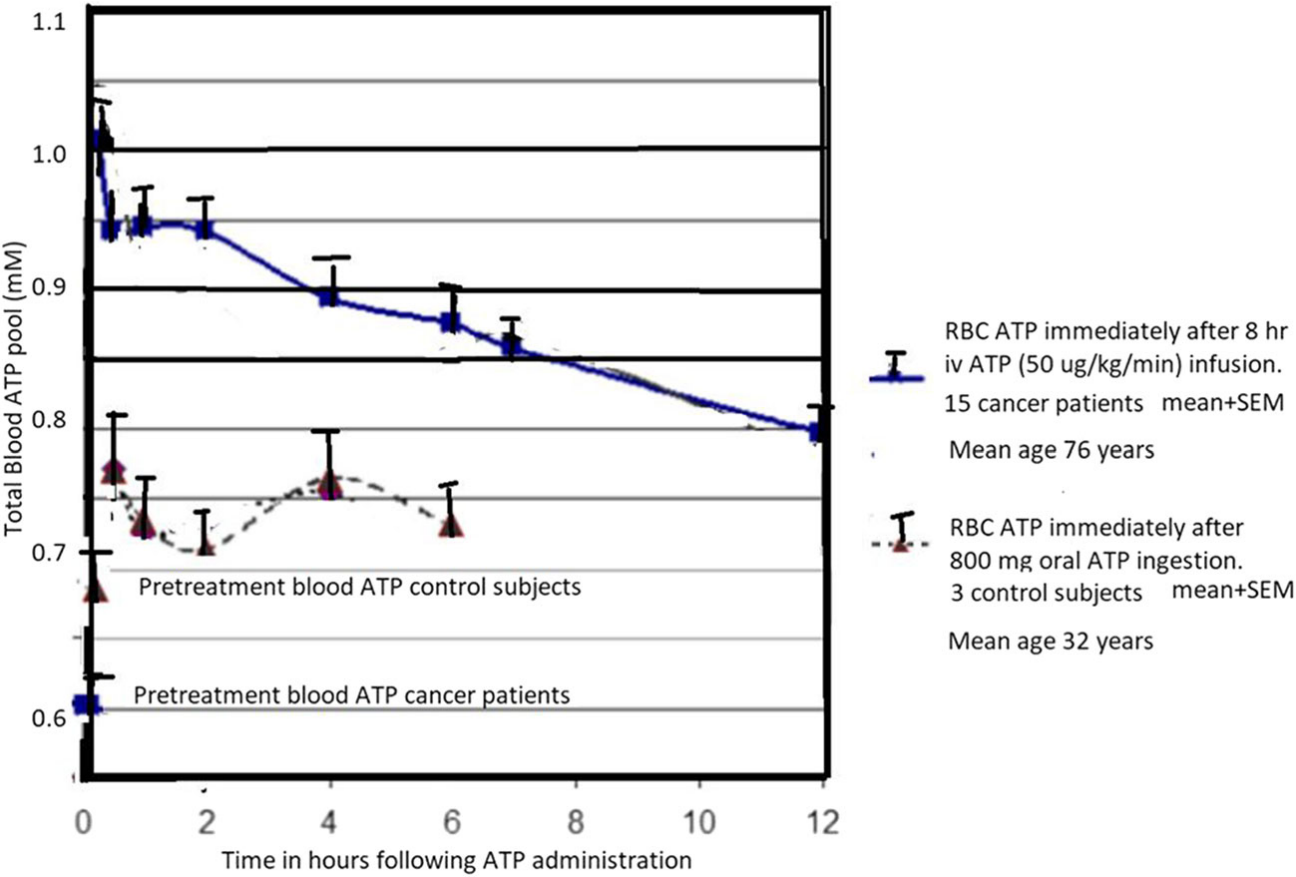
Total blood ATP pool measurements following IV ATP administration to elderly cancer patients as discussed in detail in the paper by Rapaport, Salikhova, and Abraham as compared to ATP blood pool measurements in healthy subjects taking enterically coated oral ATP at a dose of 800 mg. This higher dose was performed following the studies with Jordan et al. on athletes and demonstrates that at larger dose increase in blood ATP is detectable. In the current approach, ATP is given as non-enterically coated capsules or as ATP powder similar to the approach of Purpura et al. The figure indicates that the oral ATP on a daily dose of 800 mg is as expected less than the IV ATP administration which was well tolerated and safe. Thus, it appears that oral ATP administration is an effective method to gradually and safely replenish depleted systemic ATP pools

We are currently designing and implementing a prospective randomized trial of oral ATP versus placebo as a way to definitively confirm the benefit of replenishing systemic ATP stores in the presence of COVID-19. Pending results of such a trial, we continue to offer supplemental ATP to all patients at risk of SARS-Cov-2 exposure.

Does the worldwide implementation of SARS-CoV-2 vaccination circumvent the need for oral ATP supplementation? There appears to be multiple reasons for continued roles of oral ATP; these are as follows.

- Not all vaccinated patients develop durable (long- lasting) immunity to the SARS-CoV-2 virus. This failure becomes more pronounced in the elderly. Interestingly, both ATP and uric acid serve as adjuvants in immune stimulation following vaccine administration [22, 50, 55].
- The SARS-CoV-2 virus is now developing genetic mutations that may make the current vaccines less effective and possibly ineffective. In this case, ATP could serve as adjuvant therapy, mitigating the pathological effects of these changes [11, 21, 47].
- New viral agents will undoubtedly develop as they have in the past. ATP can function as adjuvant therapy at the present time and in future pandemics.

Falcone and coworkers demonstrated important benefits of aerosolized respiratory tract adenosine in SARS-CoV-2 acute respiratory distress due to a “cytokine storm” [17]. Adenosine modulates the A2AR receptor dependent immunomodulatory control in COVID-19 patients and dampens the SARS-CoV-2-induced cytokine storm. This therapy was subsequently applied with excellent results in a small trial of COVID-19 patients with oxygenation initiated inflammatory lung disease [15]. Oral ATP serves as a pro-drug for extracellular adenosine. We suggest that oral ATP administration generates sufficient adenosine to help avoid the COVID-19 inflammatory lung injury induced by oxygen ventilation.

## Data Availability

The datasets generated during and/or analysed during the current study are available from the corresponding author on reasonable request.

## Footnotes

1 Here and elsewhere in this communication, the acronym CF represents the homozygous presence of a mutated cystic fibrosis transmembrane conductance regulator (CFTR) gene leading to clinical cystic fibrosis. The CF phenotype results when both of the genes for the CFTR membrane proteins are mutated. (The CFTR gene is also called by some as the CFTCR gene.)

2 At present, there is no detailed information about SARS-CoV-2 infection rates and mortality rates in CFTR gene mutation heterozygotes.

3 As of the year 2001, nearly one thousand mutations of the CFTR gene had been identified. The ΔF508 mutation—the deletion of phenylalanine at position 508 of the CFTR protein—accounts for 70% of these mutations.

4 The heterozygous presence of a CFTR mutation may have produced better survival in cholera and typhoid by reducing the lethality of the diarrhea characteristic of the infection. The homozygous presence of a CFTR gene mutation may have also produced protection against cholera and typhoid, but such protection was offset by the universal lethality of the homozygous mutation.

5 Other research groups have suggested other aspects of the CF phenotype which may confer such benefit [33, 40, 45]. All of these phenomena may be operating to various degrees to confer survival benefit.

6 Solutions IRB has approved the trial with authors EHA and DMB as principal investigators.
